# DPP8/9 inhibitors activate the CARD8 inflammasome in resting lymphocytes

**DOI:** 10.1038/s41419-020-02865-4

**Published:** 2020-08-14

**Authors:** Darren C. Johnson, Marian C. Okondo, Elizabeth L. Orth, Sahana D. Rao, Hsin-Che Huang, Daniel P. Ball, Daniel A. Bachovchin

**Affiliations:** 1 Tri-Institutional PhD Program in Chemical Biology, Memorial Sloan Kettering Cancer Center, New York, NY USA; 2 Chemical Biology Program, Memorial Sloan Kettering Cancer Center, New York, NY USA; 3 Pharmacology Program of the Weill Cornell Graduate School of Medical Sciences, Memorial Sloan Kettering Cancer Center, New York, NY USA

**Keywords:** Proteases, Cell death and immune response, Immune cell death

## Abstract

Canonical inflammasomes are innate immune signaling platforms that are formed in response to intracellular pathogen-associated signals and trigger caspase-1-dependent pyroptosis. Inflammasome formation and signaling is thought to mainly occur in myeloid cells, and in particular monocytes and macrophages. Here we show that small molecule inhibitors of dipeptidyl peptidases 8 and 9 (DPP8/9), which activate the related CARD8 and NLRP1 inflammasomes, also activate pyroptosis in human and rodent resting lymphocytes. We found that both CD4^+^ and CD8^+^ T cells were particularly sensitive to these inhibitors, although the sensitivity of T cells, like macrophages, varied considerably between species. In human T cells, we show that CARD8 mediates DPP8/9 inhibitor-induced pyroptosis. Intriguingly, although activated human T cells express the key proteins known to be required for CARD8-mediated pyroptosis, these cells were completely resistant to DPP8/9 inhibitors. Overall, these data show that resting lymphoid cells can activate at least one inflammasome, revealing additional cell types and states poised to undergo rapid pyroptotic cell death in response to danger-associated signals.

## Introduction

A number of intracellular pathogen- and danger-associated signals trigger the formation of multiprotein complexes called inflammasomes^1,2^. In the “canonical” inflammasome signaling pathway, an intracellular pattern recognition receptor (PRR) detects its specific signal, oligomerizes with the adaptor protein ASC, and recruits pro-caspase-1. Pro-caspase-1 undergoes proximity induced autoproteolysis and activation on the inflammasome^3^, and then in turn cleaves and activates the pore-forming protein gasdermin D (GSDMD) and the proinflammatory cytokines pro-IL-1β and pro-IL-18^4,5^. The N-terminal fragment of GSDMD forms pores in the cellular membrane, releasing the activated cytokines and inducing pyroptotic cell death.

**Fig. 1:**
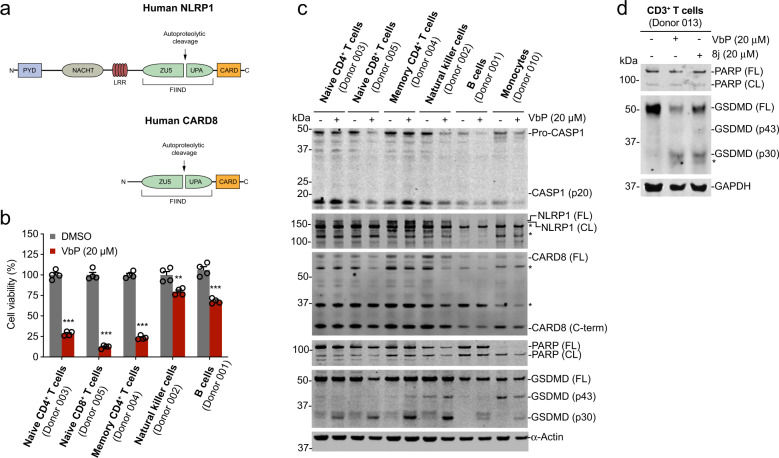
DPP8/9 inhibitors induce pyroptosis in primary lymphocytes. **a** Domain structures of human NLRP1 and CARD8. Note that the ZU5 and UPA sub-domains together are referred to as a FIIND. **b**, **c** The indicated primary cells were treated with VbP (24 h) before cell death was assessed by Cell-TiterGlo (**b**) and lysates were evaluated by immunoblotting (**c**). Data are means ± s.e.m. of four biological replicates. **d** CD3^+^ T cells were treated with VbP (20 μM, 24 h) or 8j (20 μM, 24 h) before lysates were evaluated by immunoblotting. Immunoblots are representative of three independent experiments.

NLRP1 and CARD8 are related human PRRs that form inflammasomes^6–9^. NLRP1 and CARD8 have similar C-terminal ZU5, UPA, and CARD domains, but have different N-terminal regions (Fig. 1a). NLRP1 and CARD8 both undergo autoproteolysis at the C-terminal end of their ZU5 domains, generating N- and C-terminal fragments that remain non-covalently associated^10^. Although the pathogen-associated signal (or signals) that activates NLRP1 and CARD8 has not been identified, small molecule inhibitors of the host serine proteases DPP8 and DPP9 (DPP8/9), including the nonselective DPP inhibitor Val-boroPro (VbP), were recently discovered to activate both NLRP1 and CARD8^6,9,11–13^. DPP8/9 inhibitors induce the proteasome-mediated destruction of the NLRP1 and CARD8 N-terminal fragments through an unknown mechanism^6,14^, releasing their C-terminal fragments from autoinhibition. The liberated NLRP1 UPA-CARD indirectly recruits and activates pro-caspase-1 via ASC, whereas the CARD8 UPA-CARD directly recruits and activates pro-caspase-1^3^. In addition, DPP8/9 binds directly to NLRP1 and CARD8. DPP8/9 inhibitors disrupt the NLRP1-DPP9, but not the CARD8-DPP9, interaction^9,15^, and this direct displacement also contributes to NLRP1 inflammasome formation.

Typically, inflammasomes are expressed in, and therefore studied in, monocytes and macrophages^1^. Indeed, we and others have demonstrated that DPP8/9 inhibitors induce pyroptotic cell death in many monocyte-derived cancer cell lines and primary bone marrow-derived macrophages (BMDMs), but not in cells derived from many other lineages^6,9,11,16,17^. However, DPP8/9 inhibitor-induced pyroptosis is not entirely restricted to monocytes and macrophages. For example, we found that VbP induced cell death in human CD34^+^ cord blood (hCD34^+^ CB) cells, which are a mixture of hematopoietic stem and progenitor cells, and in primary B-cell acute lymphoblastic leukemia cells^6^. Moreover, NLRP1 is expressed in human skin, and, consistent with this expression, germline mutations in NLRP1 cause skin inflammatory syndromes^7,18,19^ and DPP8/9 inhibitors induce pyroptosis in keratinocytes^9^.

In addition to these recent results, many groups have reported intriguing biological effects of dipeptidyl peptidase inhibitors in a number of cell types over the past forty years^20^. In particular, the discovery that DPP4 is highly expressed on the surface of T cells sparked extensive research into the role of DPP4 in lymphocyte activation^21,22^. In the 1980s and 1990s, DPP4 inhibitors with unknown selectivities were reported to block mitogen-induced lymphocyte proliferation using radioactive thymidine incorporation and IL-2 release assays^21,23,24^. Years later, selective inhibitors showed that these effects were in fact due to DPP8/9 inhibition^25^, but the underlying mechanism was not investigated further. In a separate set of assays in the late 1990s, VbP itself was discovered to induce a “novel apoptotic pathway” in resting, but not activated, peripheral blood mononuclear cells (PBMCs)^26^. The key VbP target was shortly thereafter determined to be “quiescent cell protease”^27^, which is now referred to as DPP7. However, selective DPP7 inhibitors developed years later did not trigger this apoptotic pathway^28^. The response of primary lymphocytes, in particular resting and activated T cells, to selective DPP8/9 inhibitors has not yet been investigated.

Here we show that DPP8/9 inhibitors induce pyroptosis in resting lymphocytes, including in naïve CD4^+^ T cells, memory CD4^+^ T cells, naïve CD8^+^ T cells, B cells, and natural killer (NK) cells. T cells were particularly sensitive to DPP8/9 inhibitors, although T-cell sensitivity, like macrophage sensitivity, varied considerably between species. As in acute myeloid leukemia (AML) cancer cell lines, we found that CARD8, and not NLRP1, mediates DPP8/9 inhibitor-induced pyroptosis in human T cells. Strikingly, activated human T cells, despite expressing the proteins involved in CARD8-mediated pyroptosis, were completely resistant to DPP8/9 inhibitors. The mechanistic basis of this resistance is unclear. Regardless, this work reveals that mammalian resting lymphocytes can respond to certain danger-associated signals via inflammasome activation and pyroptotic cell death.

## Materials and methods

### Reagents and antibodies

Compound 8j was synthesized according to previously published protocols^29^. VbP (Talabostat mesylate) was purchased from R&D Systems and was resuspended in DMSO containing 0.1% TFA to prevent cyclization. Bortezomib was purchased from LC laboratories, zVAD-FMK from Ubpbio, VX-765 from Apexbio Technology LLC, and etoposide from Enzo Life Sciences. LPS (from E. coli O111:B4) was purchased from Invivogen, Nigericin from Cayman Chemicals, Bestatin Methyl Ester from Santa Cruz, and MCC950 from AdipoGen. Antibodies used were: hCASP1 (no. 2225, Cell Signaling Technology), GAPDH (clone 14C10, Cell Signaling Technology), hGSDMD (NBP2-3342, Novus Biologicals), PARP (no. 9542, Cell Signaling Technology), hNLRP1 (AF6788, R&D Systems), hCARD8 (no. ab24186, Abcam), mGSDMD (no. ab209845, Abcam), GSDMD (no. 219800, Abcam), α-Actin (no. A4700, Sigma-Aldrich), mCD45 (no. 103108, Biolegend, FITC conjugate, clone 30-F11), mCD3 (no. 100235, Biolegend, APC conjugate, clone 17A2), rCD3 (no. 201411, Biolegend, PE conjugate, clone 1F4), rCD6 (no. 554904, BD Biosciences, FITC conjugate, clone OX-52), mCaspase-1 (AG20B-0042, Adipogen), mIL-1β (no. D4T2D, Cell Signaling Technologies), NLRC4 (no. ab201792, Abcam), and NLRP3 (no. ab210491, Abcam).

### Human primary cell isolation and culture

Isolated human primary cells were obtained from Astarte Biologics. All cells were >90% purity, as validated by flow cytometry by Astarte Biologics. T cells, B cells, and NK cells were thawed in RPMI-1640 medium supplemented with 10% FBS and cultured in RPMI-1640 medium, 10% fetal bovine serum (FBS), and 30 U/mL IL-2 (Peprotech). Monocytes were thawed in Iscove’s modified Dulbecco’s medium (IMDM) with 15% FBS and DNAse1 (1μg ml^−1^) and cultured in medium consisting of IMDM with 15% FBS, 0.1-mM 2-mercaptoethanol, 20-ng ml^−1^ G-CSF (Peprotech), 100-ng ml^−1^ SCF (Peprotech), 20-ng ml^−1^ IL-3 (Peprotech), and 50-ng ml^−1^ FLT3-Ligand (Peprotech). T cells were activated for 48 h using Human T-Activator CD3/CD28 Dynabeads for T-Cell Expansion and Activation (Gibco) according to manufacturer’s protocol.

### Mouse and rat T-cell isolation and culture

All mouse and rat experiments were performed at the MSKCC animal facility and were approved by the institutional animal care and use committee. Mouse and rat T cells were obtained from spleens harvested from female and male animals between the ages of 7 and 12 weeks. Briefly, spleens were harvested from mice or rats (male and female mixture), crushed with a syringe plunger, and strained through a 70-μm nylon cell strainer. Red blood cells (RBCs) were lysed for 4–5 min on ice in 1× RBC lysis buffer (Biolegend) and cells were centrifuged at ~320 × *g* for 5 min. Cells were washed in 10 mL of MACS buffer (1× phosphate-buffered saline [PBS], 2-mM EDTA, and 0.5% bovine serum albumin). T cells were then isolated from the cell mixture using the Pan T-Cell Isolation Kit II (Miltenyi Biotec) or rat Pan T-Cell MicroBeads (Miltenyi Biotec) according to manufacturer’s protocol. Mouse T cells were cultured in RPMI-1640 medium supplemented with 10% FBS, 1× penicillin/streptomycin (Corning), 0.01× MEM Non-Essential Amino Acids Solution (ThermoFisher Scientific), 10-mM HEPES (Gibco), 1-mM Sodium pyruvate (Gibco), 550-μM 2-mercaptoethanol (Gibco), and 30-U/mL IL-2 (Peprotech). Mouse T cells were activated for 48 h using Mouse T-Activator CD3/CD28 Dynabeads for T-Cell Expansion and Activation (Gibco) according to manufacturer’s protocol. Purity of isolated samples was confirmed by flow cytometry.

### Mouse and rat BMDM isolation and culture

Bone marrow was harvested from the femurs and tibias of 7–12 week old mice and rats. Briefly, furmurs and tibias were harvested from mice or rats (male and female) and crushed with a mortar and pestle in cold 1× PBS supplemented with 2.5% FBS. The mixture was strained through a 70-μm nylon cell strainer. RBCs were lysed for 4–5 min on ice in 1× RBC lysis buffer (Biolegend) and cells were centrifuged at 300 × *g* for 5 min at 4 °C. The cell pellet was washed in cold 1× PBS supplemented with 2.5% FBS before being strained in a 70-μm nylon cell strainer and counted. Counted cells were plated on non-tissue culture 10-cm plates at 5–10 × 10^6^ cells per plate in DMEM supplemented with 10% FBS and 15–20% L-cell media for mouse cells and 30% L-cell media for rat cells. Mouse and rat cells were incubated at 37 °C for 6 and 9 days, respectively, before assaying.

### Cell line culture

MV4;11 and RAW 264.7 cells were purchased from ATCC. MV4;11 cells were cultured in RPMI-1640 medium supplemented with 10% FBS and RAW 264.7 cells were cultured in DMEM supplemented with 10% FBS. Cell lines were tested for mycoplasma using the MycoAlert Mycoplasma Detection Kit (Lonza).

### CellTiter-Glo cell viability assay

Cells were plated (4000 cells per well) in white, 384-well clear-bottom plates (Corning) in 25-μL final volume of medium. Compounds were added using a pintool (Analytic-Jena CyBio Well Vario). Cells were incubated for the indicated timepoints at 37 °C. Assay plates were then removed from the incubator and allowed to equilibrate to ambient temperature for 30 min before adding 10 μL of CellTiter-Glo reagent (Promega). Assay plates were analyzed according to manufacturer’s protocol on a Cytation 5 Cell Imaging Multi-Mode Reader (BioTek). Relative IC_50_ values were calculated using nonlinear regression and a three-parameter dose response in GraphPad Prism version 7. Sample size was determined based on previous studies.

### T-cell immunoblotting experiments

Cells were seeded in 12-well plates at 1.5 × 10^6^–3.0 × 10^6^ cells per well. Seeded cells were treated with DMSO or compound as described for the indicated timepoint. Cells were washed twice in PBS (pH = 7.4), resuspended in PBS, and lysed by sonication. Protein concentrations were determined using the DC Protein Assay kit (Bio-Rad). The samples were separated by SDS-PAGE, immunoblotted, and visualized using the Odyssey Imaging System (LiCor).

### Mouse BMDM immunoblotting

Cells were seeded in 12-well plates at 1.5 × 10^6^ cells per well in 0.5-mL Opti-MEM. Seeded cells were treated with DMSO or compound/stimulus as described for the indicated timepoint. Media supernatant was collected and stored on ice for a supernatant immunoblot. Cells were washed twice in cold PBS (pH = 7.4) and were lysed in 0.5% NP-40 supplemented with Halt protease and phosphotase inhibitor cocktail (Thermo Scientific) for 20 min on ice. The resulting supernatant mixture was harvested, sonicated, and centrifuged at 20,000 × *g* for 10 min at 4 °C. Supernatant was transferred to a fresh tube and protein concentrations were determined using the DC Protein Assay kit (Bio-Rad). The samples were separated by SDS-PAGE, immunoblotted, and visualized using the Odyssey Imaging System (LiCor). For the media supernatant immunoblots, 500-μL methanol and 150 μL of chloroform was added to the Opti-MEM supernatant. The mixture was vortexed and centrifuged at 20,000 × *g* for 10 min at ambient temperature. The aqueous top layer was discarded and 800-μL methanol was added to the samples before they were vortexed and centrifuged. The supernatant was carefully removed from the pellets and the samples were incubated at 37 °C for 10 min with the tube lids open to dry the pellets. Fifty microliter SDS loading buffer was added to resuspend each pellet before boiling the samples for 10 min. The samples were separated by SDS-PAGE, immunoblotted, and visualized using the Odyssey Imaging System (LiCor).

### Annexin V and PI staining

Cells were seeded in 96-well plates at 0.1 × 10^6^ cells per well. The cells were treated with DMSO or the indicated compound for the described time. Following incubation, the cells were analyzed using the annexin V-FITC Apoptosis Kit (TakaraBio) according to the manufacturer’s protocol.

### CRIPSR KO from CD3^+^ T cells

CD3^+^ T cells were thawed in RPMI-1640 medium supplemented with 10% FBS and cultured in RPMI-1640 medium supplemented with 10% FBS and 30-U/mL IL-2 (Peprotech). The cells were stimulated with CD3/CD28 Dynabeads for 48 h at 37 °C. To prepare RNP complex, 50 μM of CARD8 sgRNA (Synthego, sequence: U*G*C*ACCCCGCCGGCAAUUCA + Synthego modified EZ Scaffold) was mixed with 20-μM Cas9 2NLS nuclease (Synthego) and incubated at room temperature for 10 min. The cells were removed from Dynabeads, washed in 1× PBS, and resuspended to 6.6 × 10^7^ cells/mL in buffer T from the Neon Transfection System kit. Ten microliter of cells were mixed with 1 μL of RNP complex and electroporated using the ThermoFisher Scientific Neon Transfection System at 1400 V, 10 ms, and three pulses. The cells were transferred in to a 24-well plate containing 1 mL of prewarmed culture media and was incubated for 48 h at 37 °C. The cells were pooled and incubated for 10 days before assaying for knockdown.

### Lethal factor-fused flagellin (LFn-flagellin) preparation

Five milliliter cultures of His-tagged LFn-flagellin expressing E.coli (BL21) were grown overnight at 37 °C. Five milliliter cultures were transferred to flasks containing 100 mL of media and grown to OD_600_ = 0.6–0.8. These cultures were induced with 1-mM IPTG and grown overnight at 18 °C with shaking. Cultures were centrifuged at 4000 × g for 15–30 min and were resuspended in 1× PBS (pH = 7.4, without calcium and magnesium). Cells were sonicated on ice and lysate was clarified by centrifuging at 12,000 × *g* for 30 min at 4 °C. Supernatant samples were then loaded onto a column containing Talon Metal Affinity Resin (Takara no. 635501), and LFn-flagellin was purified according to manufacturer’s protocol. Protein purification was confirmed by Coomassie stain.

### Microscopy time-lapse

CD3^+^ T cells were thawed in a 37 °C water bath. The cells were plated in a 384-well plate at 15,000 cells per well in 50 μL of culture media. Cells were treated with 2.5-μg/mL propidium iodide (PI) and immediately treated with the indicated compounds. Images of PI uptake were captured every 5 min for 18 h on a Zeiss Axio Observer.Z1 inverted wide-field microscope using 20×/0.8 NA air objective. For each well, nine positions were imaged on bright-field and red fluorescence channels at a single timepoint from a given experiment. Data were exported as raw.czi files and analyzed using custom macro written in ImageJ/FIJI.

### LDH cytotoxicity assay

Cells were seeded in 24-well plates at 0.5 × 10^6^ cells per well in 0.5 mL of culture media. Seeded cells were treated with DMSO or inflammasome stimuli as described for the indicated timepoints. Supernatants were then harvested and analyzed for LDH activity using an LDH cytotoxicity assay kit (Pierce).

### IL-1β ELISA assay

Cells were seeded in 24-well plates at 0.5 × 10^6^ cells per well in 0.5 mL of culture media. Seeded cells were treated with DMSO or inflammasome stimuli as described for the indicated timepoints. Supernatants were then harvested and analyzed for IL-1β secretion using a human IL-1-β detection kit (no. DLB50, R&D).

### Statistical analysis

Two-sided Student’s *t* tests were used for significance testing. *P* values < 0.05 were considered to be significant. Graphs and error bars represent means ± s.e.m. of independent biological experiments unless stated otherwise. For all experiments, the investigators were not blinded. Variance was similar between groups that were statistically compared. All statistical analaysis was performed using GraphPad Prism version 7. No data were excluded.

## Results

### DPP8/9 inhibition induces pyroptosis in primary human lymphocytes

We first wanted to determine the sensitivity of primary human lymphocytes to VbP. We therefore treated primary human naïve CD4^+^ T cells, naïve CD8^+^ T cells, memory CD4^+^ T cells, NK cells, and B cells with VbP for 24 h before assessing their viability using Cell-TiterGlo (Figs. 1b, S1a, b, and Table S1). We found that VbP was cytotoxic to all of these cell types. The T cells were particularly sensitive, as VbP eliminated the vast majority by 24 h. In contrast, VbP killed less than half of the NK and B-cell populations over the same time interval.

As expected, we found that VbP induced GSDMD cleavage into the pyroptotic p30 fragment without any PARP cleavage in all of these cell types (Figs. 1c and S1c), consistent with pyroptosis and not apoptosis. Furthermore, we found VbP both induced a rapid, time-dependent increase in PI uptake without an increase in annexin V^+^/PI^−^ cells, again indicative of pyroptosis (Fig. S2). We should note that we did not observe proteolytic fragments (p10 and p20) of pro-caspase-1 in these immunoblots (Fig. 1c). Although both ASC-dependent and ASC-independent inflammasomes require pro-caspase-1 autoprocessing for pyroptosis, ASC-independent inflammasomes, including the CARD8 inflammasome, typically induce so little pro-caspase-1 autoproteolysis that the cleaved p10 and p20 fragments are not observable by immunoblotting^3,30,31^. The lack of observable pro-caspase-1 processing, coupled with the high levels of CARD8 expression (Fig. 1c), suggests that VbP might activate CARD8, and not NLRP1, in these lymphocytes.

To confirm that this pyroptosis was indeed due to DPP8/9 inhibition, we next tested the specific DPP8/9 inhibitor compound 8j^29^ for the induction of cell death. As expected, 8j, like VbP, induced GSDMD cleavage into the pyroptotic p30 fragment in CD3^+^ T cells after 24 h (Figs. 1d and S1c). Similar to VbP, 8j decreased cell viability of both CD4^+^ and CD8^+^ T cells (Fig. S1b) and induced annexin V^+^/PI^+^ cells without an increase in annexin V^+^/PI^−^ cells (Fig. S2). Overall, these data confirm that DPP8/9 inhibition induces pyroptosis in resting lymphocytes.

### Activated lymphocytes are resistant to DPP8/9 inhibition

**Fig. 2:**
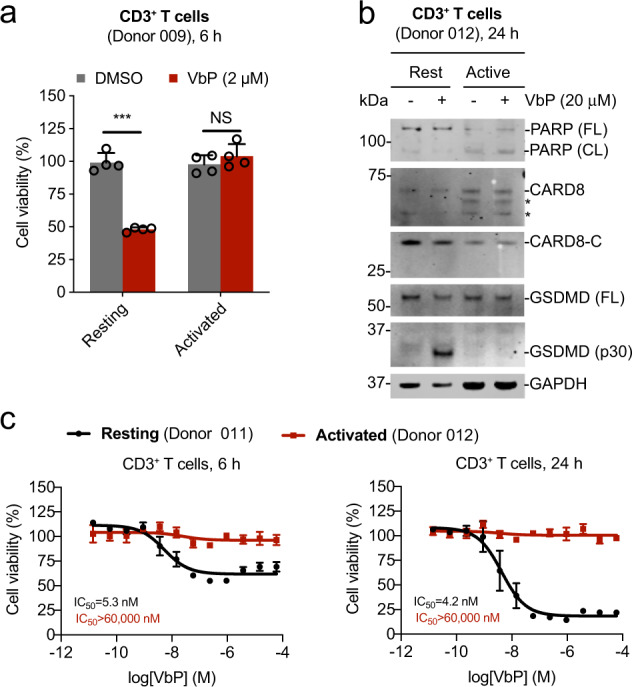
VbP induces pyroptosis in resting but not activated T cells. CD3^+^ T cells were activated with CD3/CD28 costimulation for 48 h. Resting (rest) or activated (active) T cells were then treated with the indicated concentration of VbP for the indicated time before cell viability was assessed by Cell-TiterGlo (**a**, **c**) and lysates were evaluated by immunoblotting (**b**). Data are means ± s.e.m. of four biological replicates. ***p < 0.001 by two-sided Student’s *t* test. NS not significant.

VbP was previously shown to induce considerably less death in PBMCs activated with phytohemagglutinin compared with resting PBMCs^26^. To determine if activated T cells are resistant to DPP8/9 inhibitors, we stimulated CD3^+^ T cells with CD3/CD28 Dynabeads for 48 h before treating with VbP. Strikingly, we found that activated, unlike resting T cells, were completely resistant to VbP-induced cell death (Fig. 2a). Consistent with this observation, VbP induced GSDMD cleavage in resting, but not activated, T cells (Fig. 2b). We found that resting T cells are profoundly sensitive to low doses of VbP (IC_50_ ~ 5 nM; Figs. S1a and 2c) and 8j (IC_50_ ~ 27 nM; Fig. S1a), while activated T cells remain completely resistant to high doses (>50 μM) of VbP even after 24 h (Fig. 2c). Thus, DPP8/9 inhibitors do not kill activated T cells.

### DPP8/9 inhibition activates the CARD8 inflammasome in T cells

**Fig. 3:**
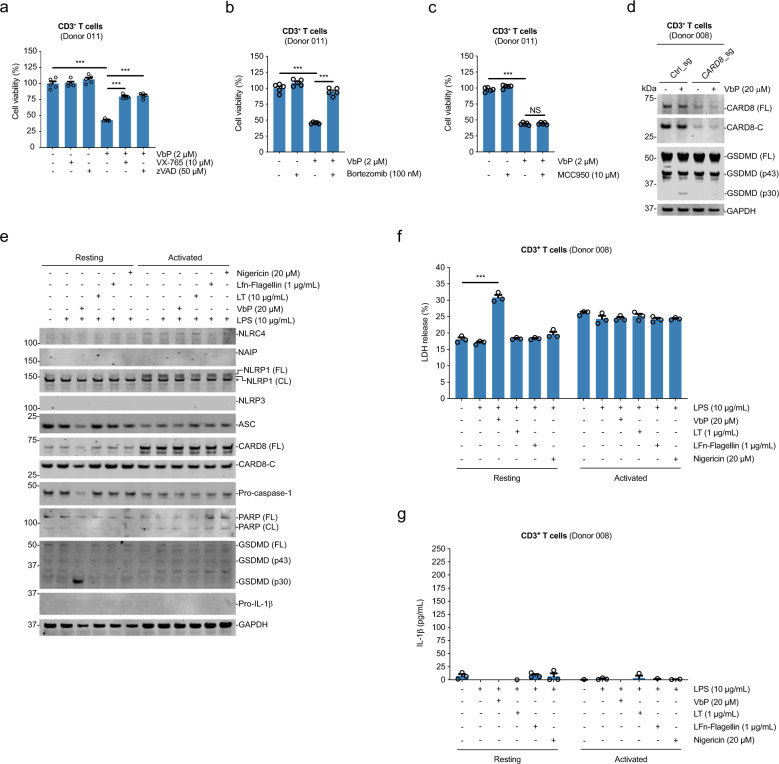
DPP8/9 inhibitors activate the CARD8 inflammasome in T cells. **a**–**c** Human primary CD3^+^ T cells were pre-treated with the indicated compounds for 30 min prior to the addition of VbP (2 μM, 6 h). Cell viability was assessed using Cell-TiterGlo. Data are means ± s.e.m. of four or five biological replicates. ***p < 0.001 by two-sided Student’s *t* test. NS not significant. **d** Immunoblots of *CARD8* KO CD3^+^ T-cell lysates treated with VbP (20 μM, 24 h). **e**, **f** CD3^+^ T cells (Donor 008) were primed with LPS (10 μg/mL, 4 h) before treatment with the indicated concentrations of VbP (6 h), LT (6 h), Lfn-flagellin (3 h), or nigericin (1 h). Lysates were then evaluated by immunoblotting (**e**) and supernatants were analyzed for LDH release (**f**) and IL-1β secretion (**g**).

We next wanted to determine the inflammasome that mediated DPP8/9 inhibitor-induced pyroptosis in resting T cells. As expected, we found that the selective caspase-1 inhibitor VX-765, the nonselective caspase inhibitor zVAD-FMK, and the proteasome inhibitor bortezomib all blocked VbP-induced cell death (Fig. 3a, b), consistent with activation of either the NLRP1 or CARD8 inflammasome. Moreover, the NLRP3 inhibitor MCC950 did not impact VbP-induced pyroptosis in CD3^+^ T cells, ruling out the involvement of the NLRP3 inflammasome (Fig. 3c).

VbP induces CARD8 activation in MV4;11 and OCI-AML2 cancer cell lines^6^. We found that resting T cells express a similar amount of CARD8 protein as these cell lines, and express even less NLRP1 (Fig. S3). The high expression of CARD8, the low expression of NLRP1, and the lack of observable VbP-induced pro-caspase-1 processing (Fig. 1c) suggested that VbP activates CARD8 in resting T cells. To test this hypothesis, we used CRISPR/Cas9 to generate *CARD8* knockout (KO) CD3^+^ T cells, and then treated these cells with VbP for 24 h. We found that VbP did not induce GSDMD cleavage in *CARD8* KO T cells (Fig. 3d), demonstrating that the CARD8 inflammasome mediates DPP8/9 inhibitor-induced pyroptosis in primary resting human T cells.

We next wanted to determine if other inflammasomes could also be activated in primary resting or activated T cells. Indeed, previous reports have demonstrated that HIV-1 and nigericin induce pyroptosis in CD4^+^ T cells derived from tonsil, spleen, and gut-associated lymphatic tissue, although not in CD4^+^ T cells derived from blood^32,33^. Here, we primed resting or activated CD3^+^ T cells with LPS for 4 h, as many PRRs require TLR priming, before the addition of VbP, nigericin, or LFn-flagellin^34^ to activate the CARD8, NLRP3, and NAIP/NLRC4 inflammasomes, respectively. We also treated cells with lethal toxin (LT), which activates the NLRP1 inflammasome in some rodent macrophages, as a negative control. We could not detect expression of NLRP3, NAIP, or IL-1β in any of these cells, and a faint NLRC4 band only in activated T cells (Fig. 3e). Consistent with the low expression of the other inflammasomes, only VbP induced GSDMD cleavage and LDH release in resting T cells without IL-1β secretion (Fig. 3e–g). Intriguingly, we observed considerably more pro-caspase-1 protein and autoproteolyzed CARD8 in resting T cells than in activated T cells, suggesting potential mechanisms of VbP resistance in the activated T cells (Figs. 2b and 3e), as discussed in detail below. Regardless, it appears that the CARD8 inflammasome is uniquely activatable in blood-derived resting T cells, although it is of course possible that other inflammasomes might be functional in different T-cell states.

### DPP8/9 inhibition induces pyroptosis in rodent T cells

**Fig. 4:**
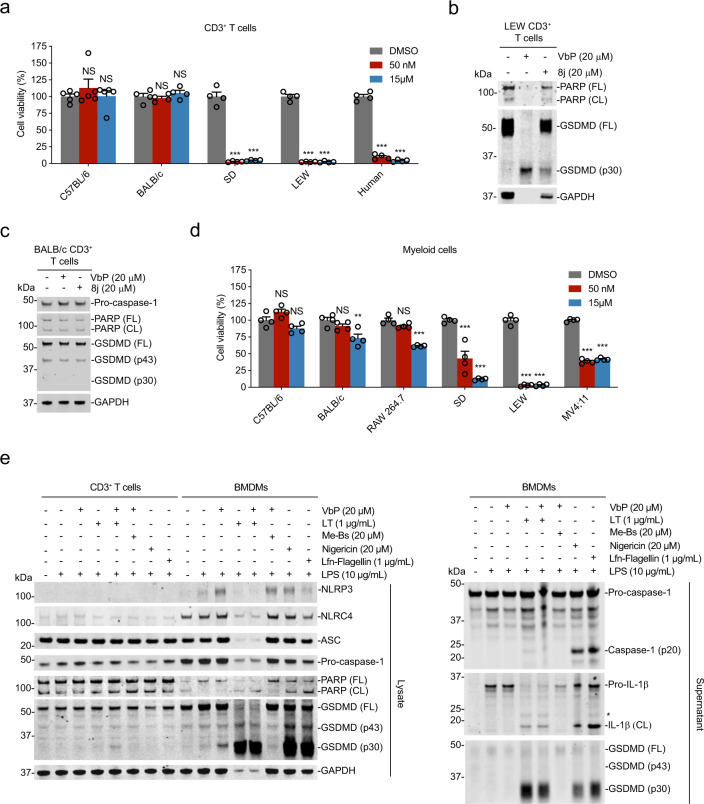
DPP8/9 inhibitors induce pyroptosis in rodent T cells. The indicated mouse, rat, and human (Donor 011) CD3^+^ T cells were treated with VbP or 8j for 24 h before cell viability was assessed by Cell-TiterGlo (**a**) and lysates were evaluated by immunoblotting (**b**, **c**). **d** C57BL/6 BMDMs, BALB/c BMDMs, SD BMDMs, LEW BMDMs, mouse RAW 264.7 cells, and human MV4;11 cells were treated with the indicated concentration of VbP for 24 h before cell viability was assessed by Cell-TiterGlo. Data are means ± s.e.m. of four or five biological replicates. ***p < 0.001, **p < 0.01 by two-sided Student’s *t* test. NS not significant. **e** BALB/c CD3^+^ T cells and BMDMs were either unprimed or primed with LPS (12 h) before being treated with VbP (6 h) methyl bestatin (Me-Bs, 6 h), LT (6 h), Lfn-Flagellin (3 h), or nigericin (1 h). Cell lysates and culture supernatants as indicated were evaluated by immunoblotting.

CARD8 is present in humans, but not in rodents. Instead, rats have one VbP-responsive NLRP1 allele and mice have two VbP-responsive NLRP1 alleles (NLRP1A and B). The rodent alleles are extremely polymorphic between inbred rodent strains, and different mouse and rat strains have strikingly different sensitivities to VbP^13,35–37^. As rodents lack CARD8, we wondered if rodent T cells could undergo DPP8/9 inhibitor-induced pyroptosis. We thus treated resting human, rat, and mouse CD3^+^ T cells with VbP for 24 h before assessing cell viability. We found that Lewis (LEW) and Sprague-Dawley (SD) rat T cells, like human T cells, were profoundly sensitive to VbP (Fig. 4a). As expected, both VbP and 8j induced GSDMD cleavage in LEW T cells (Fig. 4b). Notably, these T cells were so sensitive that little protein could be harvested from VbP-treated cells after 24 h, but, even so, a stark GSDMD p30 cleavage band could be detected (Fig. 4b). In contrast, we observed no detectable death (Fig. 4a) or GSDMD cleavage (Fig. 4c) in C57BL/6 or BALB/c mouse T cells, although BMDMs from each of these mouse strains have been reported to be responsive to VbP^12,13^.

We should note that the relative sensitivities of mouse and rat BMDMs have not been directly compared in the same experiment. Here, we found that C57BL/6 BMDMs, BALB/c BMDMs, and the BALB/c-derived RAW 246.7 cell line were all considerably less sensitive to VbP than SD BMDMs, LEW BMDMs, and human MV4;11 cancer cells (Fig. 4d). Thus, it is possible that mouse T cells are capable of undergoing pyroptosis, but are just considerably less sensitive than rat or human T cells and below the detection limit of our assays. We previously showed that LT and VbP induce synergistic cell death in LT-sensitive rodent cells. Here, we found that co-treatment of VbP and LT does indeed induce a small amount of GSDMD cleavage in mouse CD3^+^ BALB/c T cells, demonstrating that these cells are indeed capable of undergoing NLRP1-dependent pyroptosis (Fig. 4e). The nonselective aminopeptidase inhibitor methyl bestatin also synergizes with VbP in RAW 264.7 macrophages^14^, but, unlike LT, did not increase VbP-induced GSDMD cleavage in primary mouse T cells. Like human T cells, mouse T cells were resistant to nigericin and LFn-flagellin. In contrast and as expected, VbP, LT, nigericin, and LFn-flagellin all induced pyroptosis in mouse BMDMs, as evidenced by GSDMD, caspase-1, and IL-1β cleavage. However, it should again be noted that VbP induces far less GSDMD cleavage in BMDMs than the other inflammasome stimuli, including LT. Interestingly, LT induced pronounced GSDMD cleavage, but little pro-caspase-1 processing and IL-1β processing in BMDMs, perhaps suggesting the formation of a largely ASC-independent inflammasome (Fig. 4e). Overall, these data demonstrate that human, rat, and mouse T cells can all undergo DPP8/9 inhibitor-induced pyroptosis, but that mouse T cells, like mouse BMDMs, are much less sensitive to these inhibitors.

## Discussion

DPP inhibitors have shown intriguing effects on lymphocytes over the past 40 years^21,23,25,26^. Two recent studies reported that VbP induces pyroptotic cell death in human PBMCs, but the sensitive cell populations were not identified^9,11^. Here, we have now established that the inhibition of DPP8/9 activates pyroptotic cell death in a number of lymphocyte cell types. We further show that DPP8/9 inhibitor-induced pyroptosis only occurs in resting, but not activated human T cells, and that this response is mediated by the CARD8 inflammasome. Interestingly, this pathway does not involve IL-1β cleavage and release, suggesting that T cells undergo a rapid, but not necessarily hyper-inflammatory, form of lytic cell death. Overall, these data largely explain the mechanistic basis of the previously enigmatic responses induced by DPP inhibitors in lymphocytes.

It remains unclear why activated human T cells are completely resistant to DPP8/9 inhibitor-induced pyroptosis. Activated human T cells express CARD8, pro-caspase-1, and GSDMD (Fig. 3e), the three key proteins involved in this pyroptotic pathway. In fact, the ectopic expression of these proteins in VbP-resistant HEK 293 T cells renders them sensitive to DPP8/9 inhibitors^6^. It appears that activated T cells express slightly less pro-caspase-1 than resting T cells (Fig. 3e), but it would be surprising if this subtle difference in expression could account for such a drastic difference in sensitivity. Interestingly, we observed that CARD8 undergoes considerably less autoproteolysis in activated T cells than in resting T cells (Figs. 2b and 3e), indicating that less “functional” CARD8 exists in activated T cells. As such, we speculate that the extent of CARD8 autoproteolysis may be tunable, and perhaps a certain level of autoprocessing is required to enable pyroptosis. Alternatively, an entirely unknown regulatory mechanism may prevent CARD8 inflammasome formation in activated T cells. The mechanistic basis of the sensitivity difference between resting and activated T cells warrants future study.

Our results also further underscore the remarkable differences in DPP8/9 inhibitor sensitivities between strains and species, in particular revealing the extraordinary disparity between rats and mice (Fig. 4). Interestingly, *Toxoplasma gondii* (*T. gondii*) infection also activates rodent NLRP1 inflammasomes, and the relative sensitivities of rodent macrophages to *T. gondii* and DPP8/9 inhibitors are very similar^13,38,39^. We hypothesize that the different rodent NLRP1 alleles may have simply evolved to sense different levels of a specific danger signal induced by *T. gondii* and DPP8/9 inhibition. However, it is plausible that additional factors, potentially including the mechanism that inhibits inflammasome signaling in activated T cells, are also restraining NLRP1 activation in mouse cells.

More generally, this work is, to our knowledge, the first report of caspase-1-dependent pyroptosis occurring in resting blood-derived lymphocytes, revealing that canonical inflammasome signaling pathways are not restricted to myeloid cells in blood. On that note, these data suggest that cancer cells arising from a number of lymphocyte lineages, like AML cells^6^, might be sensitive to DPP8/9 inhibitors, but also indicates more potential for toxicity, particularly in humans. Projecting forward, we expect that these results will not only lead to a greater understanding of the processes that help restrain infection, but will also reveal new and exciting opportunities to modulate inflammasome activation for therapeutic benefit.

## Supplementary information

Supplemental Material

Fig S1

Fig S2

Fig S3
