# Supplementary material for: DPP8/9 inhibitors activate the CARD8 inflammasome in resting lymphocytes
Source: Cell Death Dis. 2020 Aug 14;11(8):628. doi: 10.1038/s41419-020-02865-4 (PMC7428001; doi:10.1038/s41419-020-02865-4)
Supplement: Supplementary file 1 — Supplemental Material [file 41419_2020_2865_MOESM1_ESM.docx]

**Table S1: Primary human samples used in this study.**

| **Cell type** | **Sex** | **Age** | **Donor ID** |
| --- | --- | --- | --- |
| Human CD3^+^ T cells (negatively selected) | Male | 62 | 011 |
| Human CD3^+^ T cells (negatively selected) | Male | 22 | 012 |
| Human CD3^+^ T cells (negatively selected) | Female | 42 | 013 |
| Human CD3^+^ T cells (negatively selected) | Male | 53 | 014 |
| Human CD3^+^ T cells (negatively selected) | Male | 48 | 008 |
| Human CD3^+^ T cells (negatively selected) | Female | 52 | 009 |
| Human CD3^+^ T cells (negatively selected) | Male | 20 | 015 |
| Human CD4^+^ T cells (negatively selected) | Male | 41 | 006 |
| Human CD8^+^ T cells (negatively selected) | Male | 24 | 007 |
| Human naïve CD4^+^ T cells | Male | 22 | 003 |
| Human naïve CD8^+^ T cells (negatively selected) | Female | 45 | 005 |
| Human Memory CD4^+^ T Cells | Male | 39 | 004 |
| Human B cells (negatively selected) | Male | 50 | 001 |
| Human NK cells (negatively selected) | N/A | 56 | 002 |
| Human Monocytes (negatively selected) | Female | 45 | 010 |

**Fig S1. DPP8/9 inhibition kills CD4^+^ and CD8^+^ T cells.** (**a**) Human resting CD3^+^ T cells were treated with the indicated concentrations of VbP or 8j for 24 h before cell viability was assessed using Cell-TiterGlo. Data are means ± s.e.m. of 4 biological replicates. (**b**) Human resting CD4^+^ and CD8^+^ T cells were treated with VbP or Compound 8j for 24 h before cell viability was assessed using Cell-TiterGlo. Data are means ± s.e.m. of five biological replicates. ***P < 0.001 by two-sided Student’s *t*-test. (**c**) CD3^+^ T cells were treated with VbP (2 μM, 24 h), 8j (2 μM, 24h), or etoposide (50 μM, 24 h) before lysates were evaluated by immunoblotting. Etoposide was used as an apoptosis-inducing control. Immunoblots are representative of 3 independent experiments.

**Fig S2. DPP8/9 inhibitors induce pyroptotic cell death in T cells.** (**a**) CD3^+^ T cells were treated with VbP (20 μM) or 8j (20 μM) and immediately stained with PI. PI uptake was recorded for 18 h on a ZEN microscope at 20× magnification and 0.8 NA. (**b-g**) CD3^+^ T cells were treated with the indicated drugs for 6 h (**b**-**d**) or 24 h (**e**-**g**). Cells were then stained with Annexin V and PI and were analyzed by flow cytometry. FACS dot plots in **b** and **e** are representative of 3-5 biological replicates. Panels **c** and **d** are quantifications of panel **b**, and panels **f** and **g** are quantifications of panel **e**. Data are means ± s.e.m. of 3-5 biological replicates. **p<0.01,***p<0.001 < 0.001 by two-sided Student’s *t*-test. NS, not significant. Etoposide was used as a control apoptosis-inducing agent.

**Fig S3. CARD8 and NLRP1 protein expression in T cells and AML cells.** Immunoblots of whole cell lysates from human CD3^+^ T cells (Donor 012), MV4;11 cells, and OCI-AML2 cells. Immunoblots are representative of >3 independent experiments.
