# Supplementary figures and images for: DPP8/9 inhibitors activate the CARD8 inflammasome in resting lymphocytes
Source: Cell Death Dis. 2020 Aug 14;11(8):628. doi: 10.1038/s41419-020-02865-4 (PMC7428001; doi:10.1038/s41419-020-02865-4)

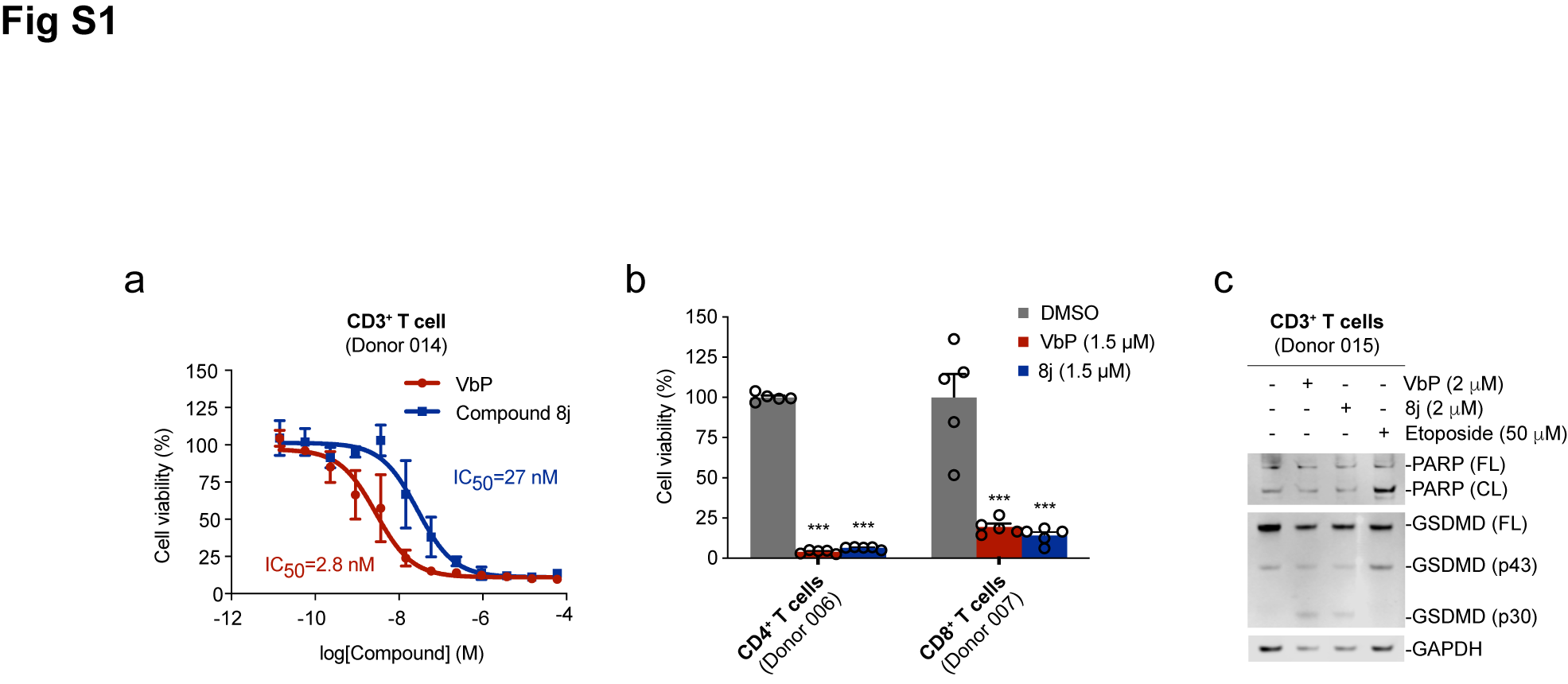

Supplement: Supplementary file 2 — Fig S1 [file 41419_2020_2865_MOESM2_ESM.tif]

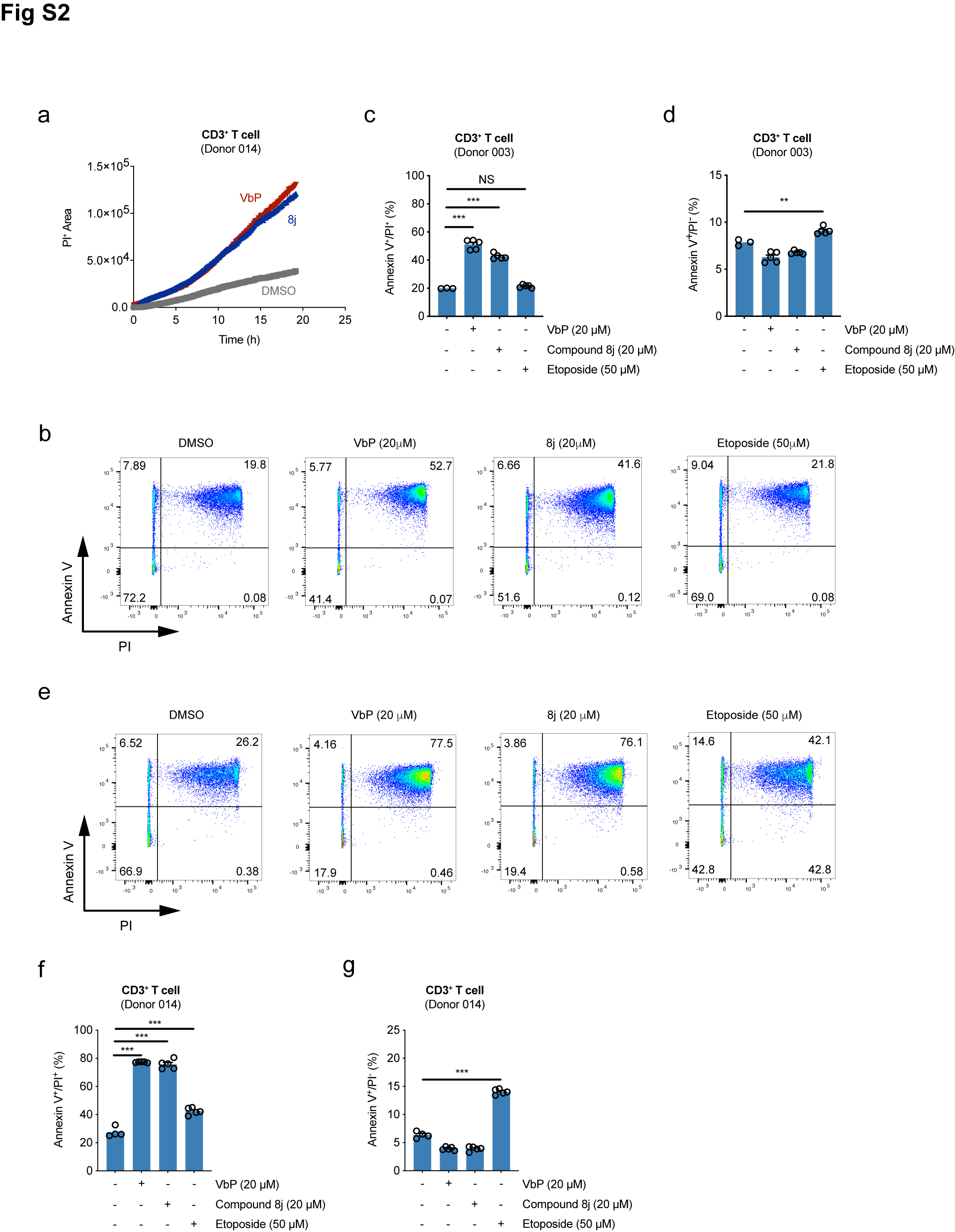

Supplement: Supplementary file 3 — Fig S2 [file 41419_2020_2865_MOESM3_ESM.tif]

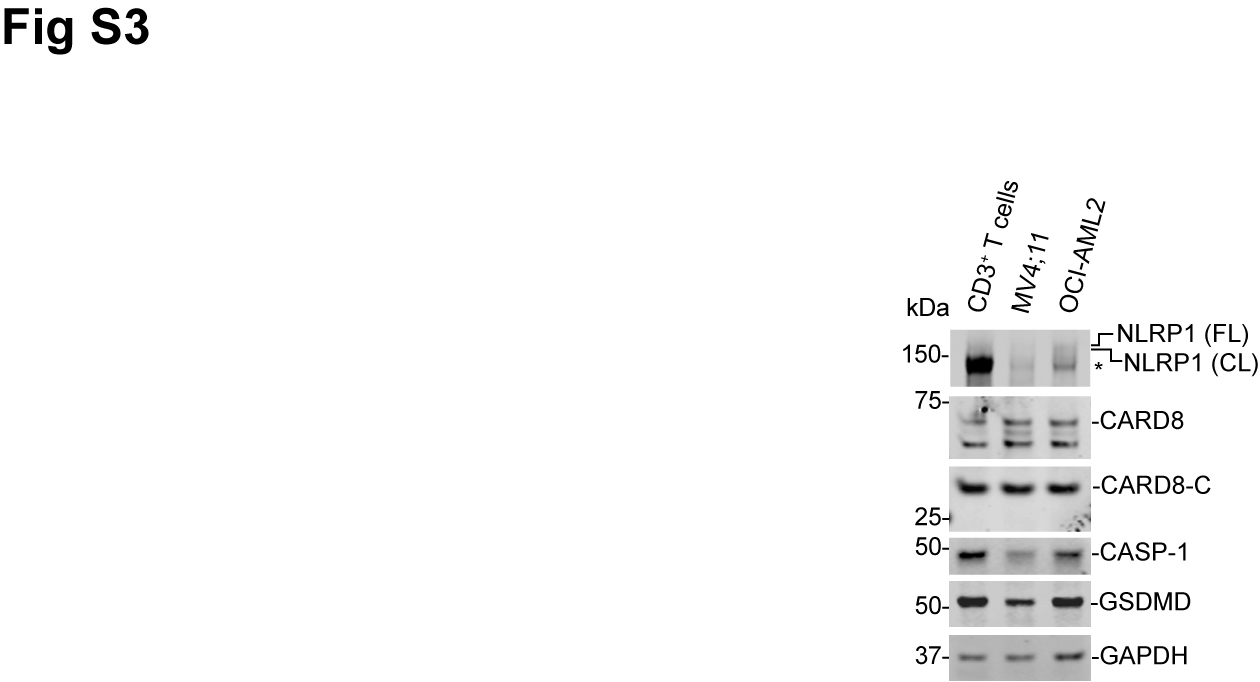

Supplement: Supplementary file 4 — Fig S3 [file 41419_2020_2865_MOESM4_ESM.tif]
